# Supplementary material for: Sequence signatures of two IGHV3-53/3-66 public clonotypes to SARS-CoV-2 receptor binding domain
Source: bioRxiv. 2021 Jan 27:2021.01.26.428356. Preprint. [Version 1] doi: 10.1101/2021.01.26.428356 (PMC7852275; doi:10.1101/2021.01.26.428356)
Supplement: Supplement 1 [file media-1.pdf]

Supplementary Figures

a

CC12.1 (Rogers et al. 2020) – Clonotype 1

```
                    C  A  R  D  L  D  V  Y  G  L  D  V  W
                   TGTGCGAGAGATTTAGATGTCTACGGTTTGGACGTCTGG
Germline sequence: TGTGCGAGAGA   AGATG CTACGGTATGGACGTCTGG
                     IGHV3-53  IGHD5-24       IGHJ6
```

COV072_P3_HC_80-P1369 (Robbiani et al. 2020) – Clonotype 1

```
                    C  A  R  D  L  G  D  Y  G  M  D  V  W
                   TGTGCGAGAGATCTGGGGGACTACGGAATGGACGTCTGG
Germline sequence: TGTGCGAGAGA  TGGGGGACTACGGTATGGACGTCTGG
                     IGHV3-66  IGHD3-16       IGHJ6
```

COV2-2080 (Zost et al. 2020) – Clonotype 1

```
                    C  A  R  D  L  V  T  Y  G  L  D  V  W
                   TGTGCGAGAGATCTCGTGACTTACGGTTTGGACGTCTGG
Germline sequence: TGTGCGAGAGA    GTGACTTACGGTATGGACGTCTGG
                     IGHV3-66    IGHD2-21     IGHJ6
```

1-20 (Liu et al. 2020) – Clonotype 1

```
                    C  A  R  D  L  F  Y  Y  G  M  D  V  W
                   TGTGCGAGAGATCTATTTTACTACGGTATGGACGTCTGG
Germline sequence: TGTGCGAGAGA CTATT TACTACGGTATGGACGTCTGG
                     IGHV3-53 IGHD2-21        IGHJ6
```

b

CC12.1 (Rogers et al. 2020) – Clonotype 2

```
                    C  A  R  D  F  G  D  F  Y  F  D  Y  W
                   TGTGCGAGAGACTTCGGTGACTTCTACTTTGACTACTGG
Germline sequence: TGTGCGAGAGA   CGGTGACT CTACTTTGACTACTGG
                     IGHV3-53    IGHD4-17       IGHJ4
```

COVD21_P1_HC_A10-p1369 (Robbiani et al. 2020) – Clonotype 2

```
                    C  A  R  D  Y  G  D  F  Y  F  D  Y  W
                   TGTGCGAGGGATTACGGTGACTTCTACTTTGACTACTGG
Germline sequence: TGTGCGAGAGA TACGGTGACT CTACTTTGACTACTGG
                     IGHV3-53   IGHD4-17        IGHJ4
```

HbnC3t1p2_B10 (Kreer et al. 2020) – Clonotype 2

```
                    C  A  R  D  Y  G  D  Y  F  F  D  Y  W
                   TGTGCGAGAGATTATGGTGACTACTTCTTTGACTACTGG
Germline sequence: TGTGCGAGAGA   TGGTGACTACTACTTTGACTACTGG
                     IGHV3-66    IGHD2-21       IGHJ4
```

Supplementary Figure 1. Putative germline sequences for CDR H3 in representative antibodies from clonotypes 1 and 2. Amino acid and nucleotide sequences of the V-D-J junction of **(a)** four clonotype 1 antibodies from different studies, and **(b)** three clonotype 2 antibodies from different studies, are shown. Putative germline sequences and segments are indicated. Somatically mutated nucleotides are underlined. Intervening spaces at the V-D and D-J junctions are N-nucleotide additions.

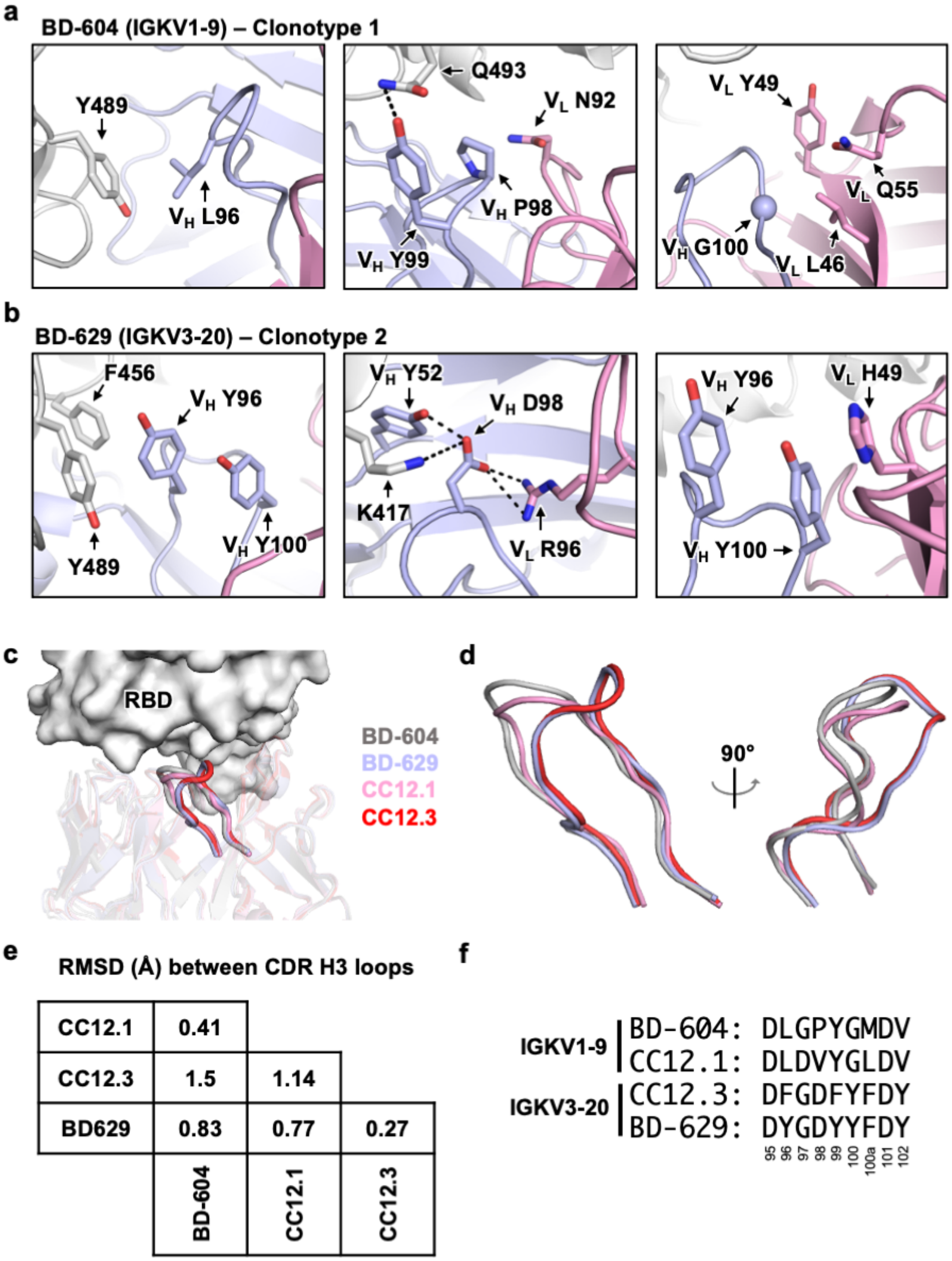

Supplementary Figure 2. Interactions between CDR H3 and the light chain of two clonotypes. (a) Interactions of L96, V98 and G100 (Kabat numbering) in CDR H3 of BD-604 (PDB 7CH4) with the IGKV1-9 light chain, and SARS-CoV-2 RBD. **(b)** Interactions of Y96, D98 and Y100 (Kabat numbering) in CDR H3 of BD-629 (PDB 7CH5) with the IGKV3-20 light chain,

and SARS-CoV-2 RBD. Gray: RBD; light blue: heavy chain; pink: light chain. **(c-d)** Structural alignment of four antibodies highlighting the structural similarities and differences of the CDR H3 loop. Gray: BD-604; light blue: BD-629; pink: CC12.1; red: CC12.3. **(c)** An overall view with RBD as white surface and antibodies as cartoon representations. The antibodies are semi-transparent except for the CDR H3 loops. **(d)** Zoomed-in views of the CDR H3 loops. **(e)** Root-mean-square deviation (RMSD) are shown for the atomic positions of the CDR H3 loops from different antibodies. **(f)** Sequences of CDR H3 loops from different antibodies are shown. Amino acids are labeled according to Kabat numbering.

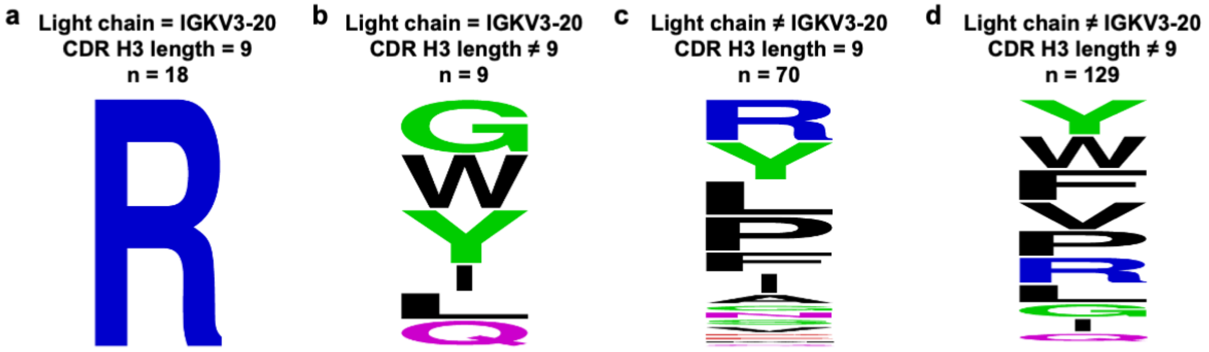

Supplementary Figure 3. Sequence logos of residue 96 in CDR L3 of IGHV3-53/3-66 RBD antibodies. (a) IGKV3-20 as light chain and CDR H3 length of 9 (n = 18), **(b)** IGKV3-20 as light chain and CDR H3 length not 9 (n = 9), **(c)** non-IGKV3-20 as light chain and CDR H3 length of 9 (n = 70), and **(d)** non-IGKV3-20 as light chain and CDR H3 length not equal to 9 (n = 129).

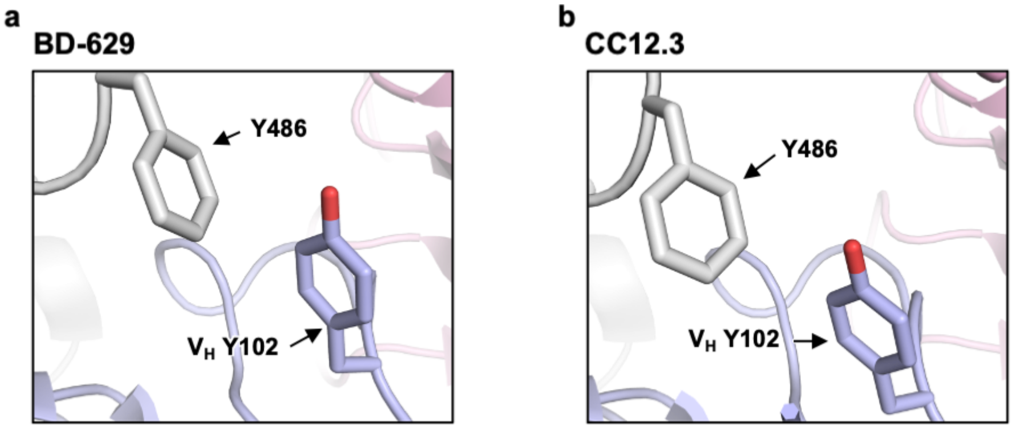

Supplementary Figure 4. Interactions between Y102 of CDR H3 loop of class 2 antibodies and the RBD of the S protein. (a) Interaction of Y102 (Kabat numbering) in CDR H3 of BD-629 (PDB 7CH5) with the RBD of the S protein. **(b)** Interaction of Y102 (Kabat numbering) in CDR H3 of CC12.3 (PDB 6XC4) with the RBD of the S protein.

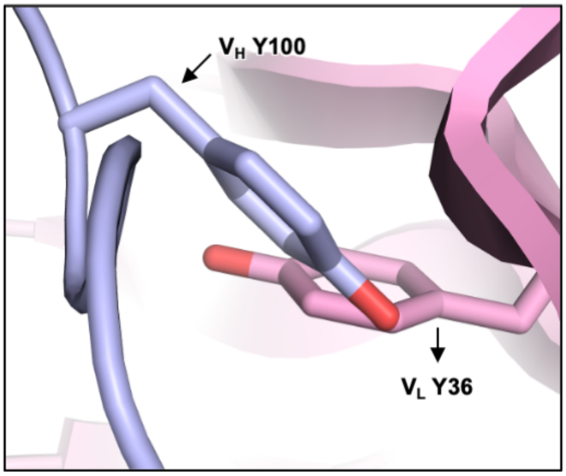

Supplementary Figure 5. V$_H$ Y100 would clash with the light chain. Rosetta modeling[1] of G100 of CC12.1 (PDB 6XC2), a clonotype 1 antibody, to Y100 with a fixed backbone indicates a clash of V$_H$ Y100 with the V$_L$ Y36.

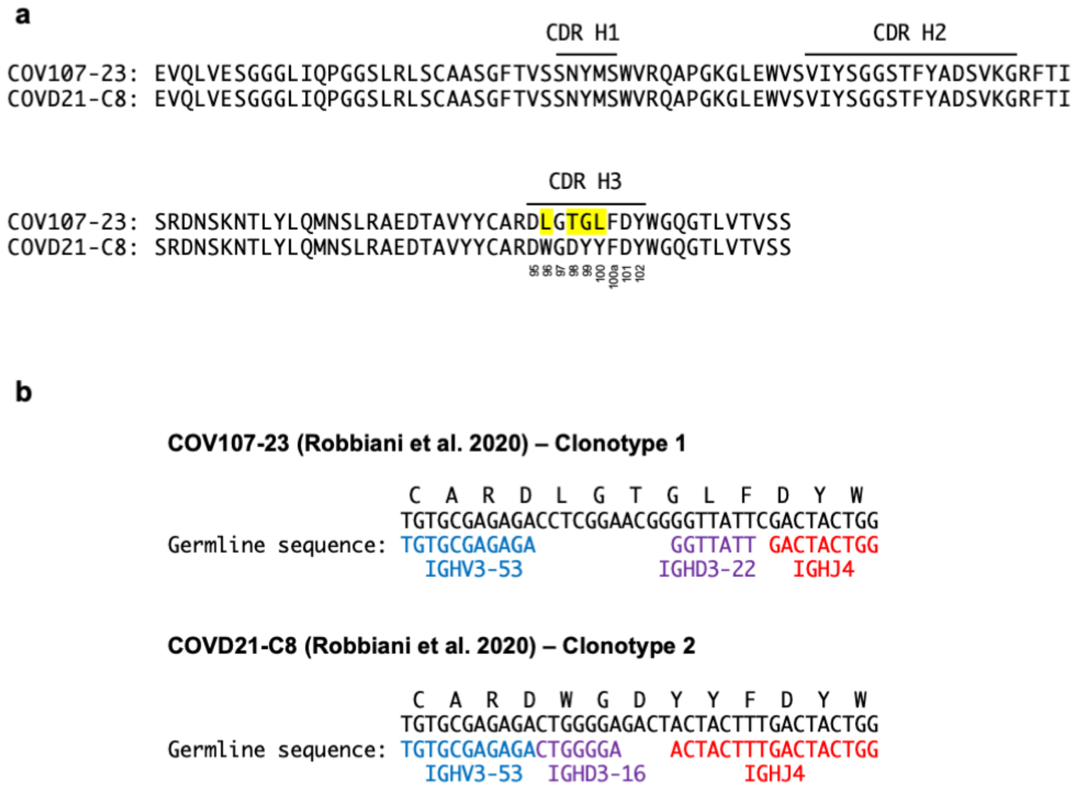

Supplementary Figure 6. Putative germline sequences for CDR H3 in representative antibodies from clonotypes 1 and 2. (a) The heavy chains of COV107-23 (clonotype 1) and COVD21-C8 (clonotype 2) RBD antibodies from Robbiani et al.[2] are aligned as shown. CDRs are annotated based on Kabat numbering. **(b)** Amino acid and nucleotide sequences of the V-D-J junction of COV107-23 and COVD21-C8 are shown. Putative germline sequences and segments are indicated. COV107-23 and COVD21-C8 are named as COV107_Plate2_HC_23-P1369 and COVD21_P1_HC_C8-p1369, respectively, in Supplementary Table 1 of Robbiani et al.[2]

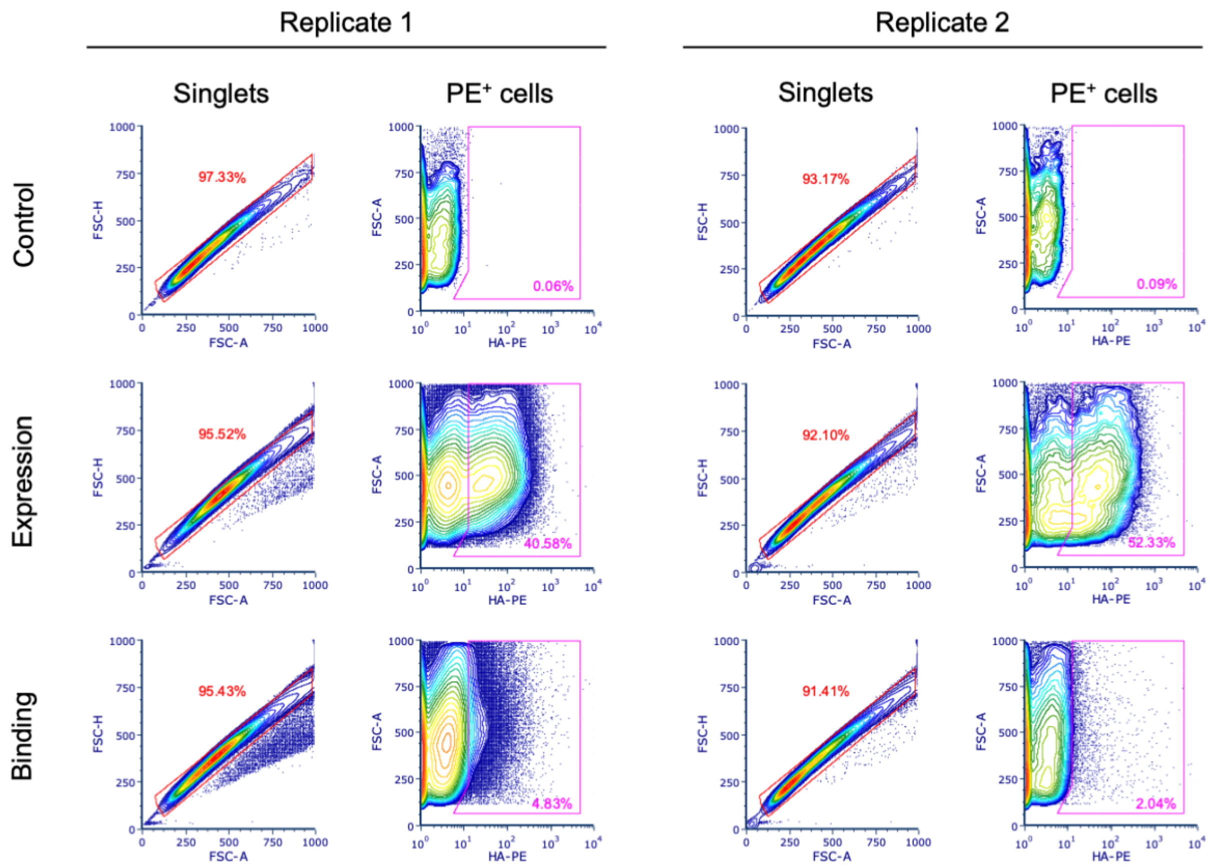

Supplementary Figure 7. Density plots of fluorescence-activated cell sorting of yeast cells. Cells from the yeast display library were sorted based on signal of PE. PE-positive singlets were collected. In the first replicate, the singlet gate was unrestricted, while in the second replicate, the singlet gate was restricted to collect 100,000 cells.

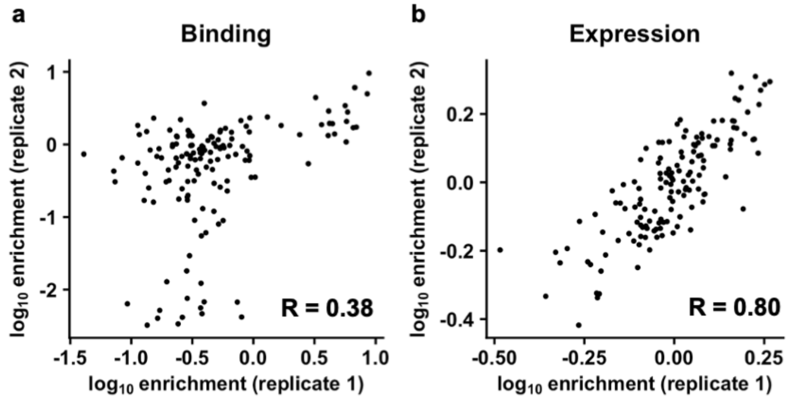

Supplementary Figure 8. Correlation between biological replicates of the yeast display selection experiment for 143 CDR H3 variants in the B38 antibody. (a) Correlation of $\log_{10}$ enrichment in binding between biological replicates is shown. **(b)** Correlation of $\log_{10}$ enrichment in expression between biological replicates is shown. The Pearson correlation (R) is indicated.

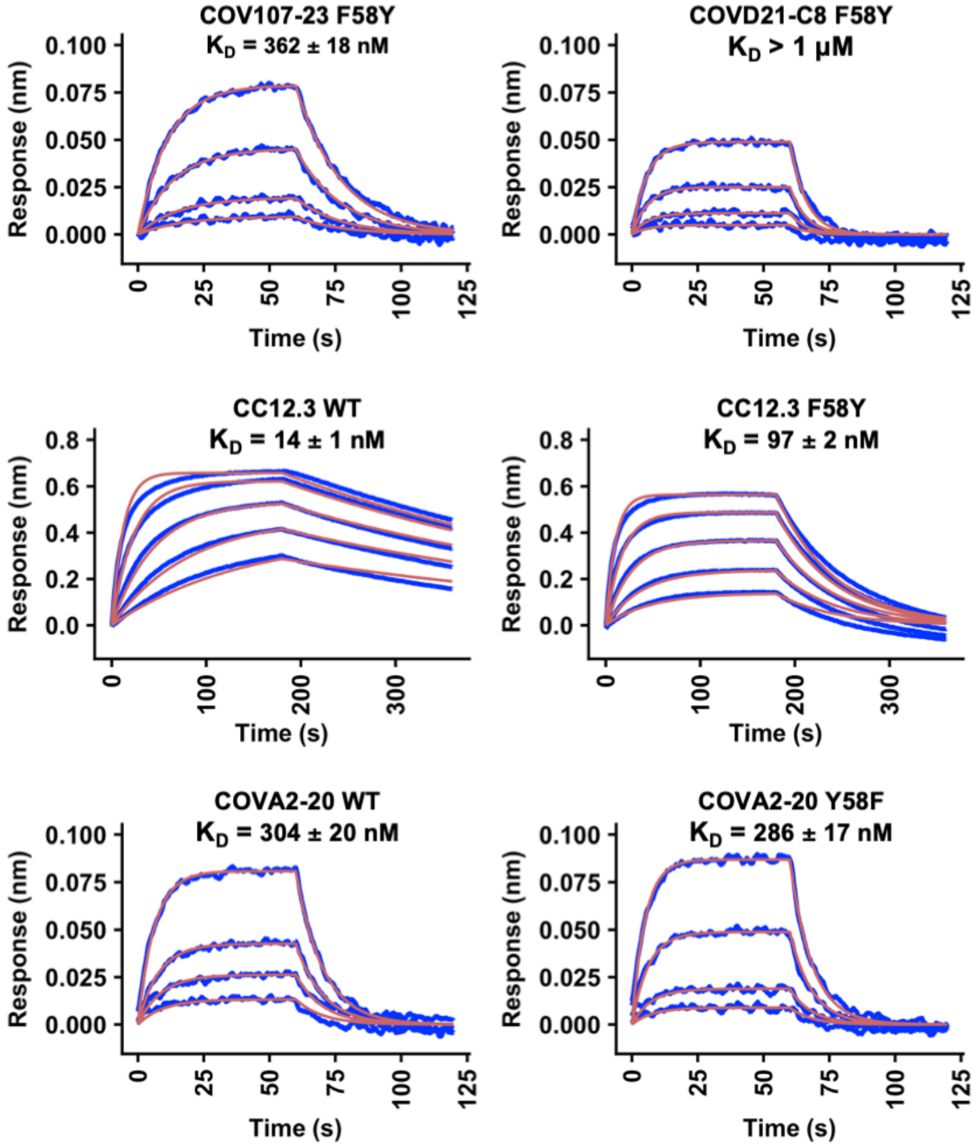

Supplementary Figure 9. Sensorgrams for binding of Fabs to SARS-CoV-2 RBD. Binding kinetics of different Fabs against recombinant SARS-CoV-2 RBD were measured by biolayer interferometry (BLI). Y-axis represents the response. Blue lines represent the response curve and red lines represent a 1:1 binding model. Binding kinetics were measured for four to five Fab concentrations.

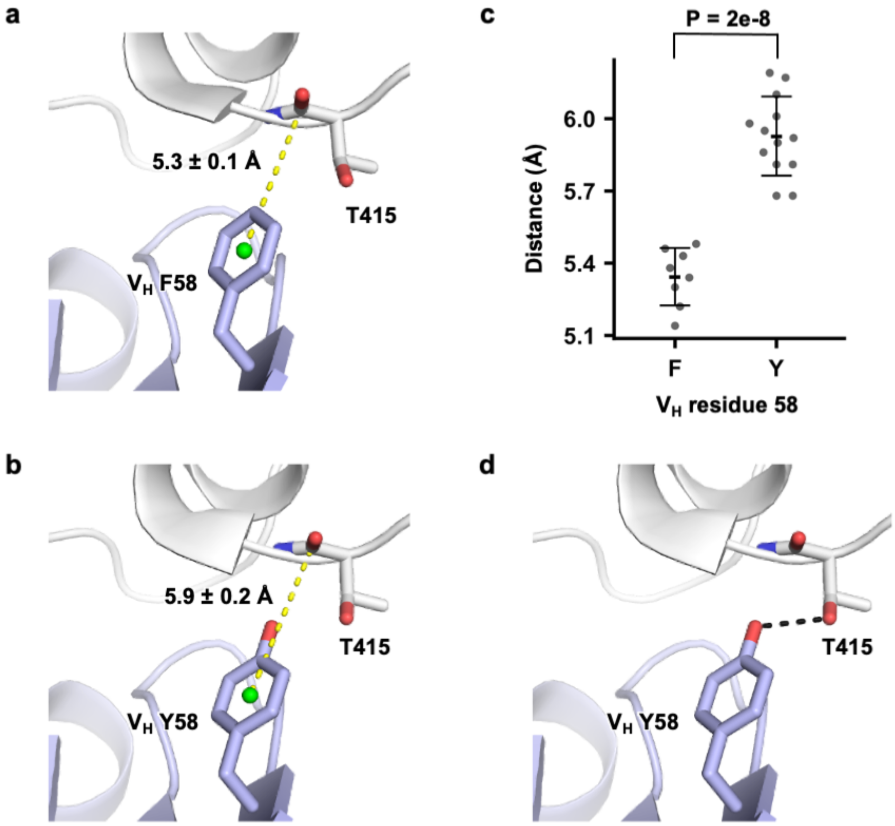

Supplementary Figure 10. Structural analysis of the Y58F mutation. (a) The distance between the centroid of the aromatic ring of V_H F58 and the backbone carbon atom of RBD T415 was measured for seven IGHV3-53/66 RBD antibodies with Y58F mutation, namely P4A1 (PDB 7CJF), CC12.1 (PDB 6XC3), CC12.3 (PDB 6XC4), C102 (PDB 7K8M), BD-604 (PDB 7CH4), COVA2-04 (PDB 7JMO), and CB6 (PDB 7C01). (n.b., there are two copies of CC12.3-RBD complex in PDB 6XC4 and both copies were analyzed). The mean and standard deviation are indicated. The centroid of the aromatic ring is represented by the green sphere. **(b)** Same as panel **a**, except for nine IGHV3-53/3-66 RBD antibodies without a Y58F mutation, namely CV30 (PDB 6XE1), STE90-C11 (PDB 7B3O), B38 (PDB 7BZ5), P2C-1F11 (PDB 7CDI), C1A-B3 (PDB 7KFW), BD-629 (PDB 7CH5), C1A-F10 (PDB 7KFY), C1A-B12 (PDB 7KFV), and C1A-C2 (PDB 7KFX). (n.b., there are three copies of C1A-B3-RBD complex in PDB 7KFW and three copies of C1A-B12-RBD complex in PDB 7KFV, all of which were analyzed). **(c)** The distance data reported

in panels **a** and **b** are shown. **(d)** The hydroxyl group of V_H Y58 can form a H-bonds (black dashed line) with RBD T415. The hydrogen bond is identified by PISA (https://www.ebi.ac.uk/pdbe/pisa/)[3].

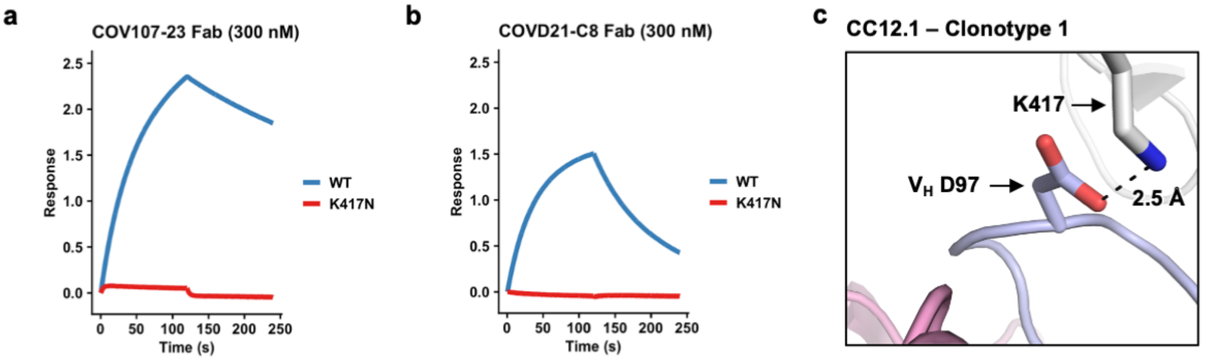

Supplementary Figure 11. Clonotypes 1 and 2 strongly bind to wild-type SARS-CoV-2 RBD but not to SARS-CoV-2 K417N RBD mutant. Binding kinetics of **(a)** COV107-23 (clonotype 1) and **(b)** COVD21-C8 (clonotype 2) Fabs to recombinant SARS-CoV-2 RBD were measured by biolayer interferometry. Y-axis represents the response. Blue and red lines represent the response curve for binding of Fabs to wild-type (WT) and K417N RBD mutant, respectively. **(c)** Electrostatic interaction between V_H D97 of CC12.1 (PDB 6XC2), a clonotype 1 antibody, with K417 RBD.

Supplementary Tables

Supplementary Table 1. IGHV3-53/3-66 RBD antibodies. List of 214 published IGHV3-53/3-66 RBD antibodies obtained from convalescent patients infected with SARS-CoV-2.

Supplementary Table 2. X-ray data collection and refinement statistics

**Data collection**		
	COVA107-23 WT	COVA107-23 HC + COVD21-C8 LC
Beamline	SSRL 12-1	SSRL 12-1
Wavelength (Å)	0.97946	0.97946
Space group	P 2	P 1
Unit cell parameters		
a, b, c (Å)	79.4, 74.8, 172.7	73.3, 78.4, 91.6
α, β, γ (°)	90, 99.4, 90	100.1, 112.1, 92.8
Resolution (Å) [a]	50.0–1.98 (2.01–1.98)	50.0–3.30 (3.40–3.30)
Unique reflections [a]	130,795 (12,808)	24,557 (2,165)
Redundancy [a]	3.5 (2.9)	1.7 (1.6)
Completeness (%) [a]	94.3 (94.5)	82.3 (70.7)
$\langle I/\sigma_I \rangle$ [a]	13.8 (1.0)	2.7 (1.0)
R_{sym}[b] (%) [a]	14.0 (>100)	29.9 (70.9)
R_{pim}[b] (%) [a]	5.7 (64.0)	18.8 (46.4)
$CC_{1/2}$[c] (%) [a]	99.7 (66.3)	93.4 (72.7)
**Refinement statistics**		
Resolution (Å)	30.7–1.98	35.9–3.30
Reflections (work)	130,416	24,493
Reflections (test)	6,632	1,186
R_{cryst}[d] / R_{free}[e] (%)	21.4/26.3	29.8/31.9
No. of atoms	14,029	12,881
Macromolecules	12,888	12,881
Solvent	1,137	0
Average *B*-value ($Å^2$)	38	59
Macromolecules	38	59
Solvent	41	N/A
Wilson *B*-value ($Å^2$)	30	54
**RMSD from ideal geometry**		
Bond length (Å)	0.012	0.014
Bond angle (°)	1.29	0.74
**Ramachandran statistics (%)**		
Favored	95.5	95.2
Outliers	0.06	0.00
**PDB code**	Pending	Pending

[a] Numbers in parentheses refer to the highest resolution shell.

[b] $R_{sym} = \Sigma_{hkl} \Sigma_i | I_{hkl,i} - \langle I_{hkl} \rangle | / \Sigma_{hkl} \Sigma_i I_{hkl,i}$ and $R_{pim} = \Sigma_{hkl} (1/(n-1))^{1/2} \Sigma_i | I_{hkl,i} - \langle I_{hkl} \rangle | / \Sigma_{hkl} \Sigma_i I_{hkl,i}$, where $I_{hkl,i}$ is the scaled intensity of the ith measurement of reflection h, k, l, $\langle I_{hkl} \rangle$ is the average intensity for that reflection, and n is the redundancy.

[c] $CC_{1/2}$ = Pearson correlation coefficient between two random half datasets.

[d] $R_{cryst} = \Sigma_{hkl} | F_o - F_c | / \Sigma_{hkl} | F_o | \times 100$, where F_o and F_c are the observed and calculated structure factors, respectively.

[e] R_{free} was calculated as for R_{cryst}, but on a test set comprising 5% of the data excluded from refinement.

Supplementary Table 3. Oligonucleotide sequences encoding CDR H3 loops. List of 143 oligonucleotides encoding CDR H3 loops used in generation of a yeast antibody display library.

Supplementary Table 4. Read count and enrichment level of each CDR H3 variant in the yeast display screen.

Supplementary References

1. Kellogg, E. H., Leaver-Fay, A. & Baker, D. Role of conformational sampling in computing mutation-induced changes in protein structure and stability. *Proteins: Struct. Funct. Bioinform.* **79**, 830-838 (2011).

2. Robbiani, D. F. *et al.* Convergent antibody responses to SARS-CoV-2 in convalescent individuals. *Nature* **584**, 437-442 (2020).

3. Krissinel, E. & Henrick, K. Inference of macromolecular assemblies from crystalline state. *J. Mol. Biol.* **372**, 774-797 (2007).
